# Validity and reliability of the Malay version of sleep apnea quality of life index – preliminary results

**DOI:** 10.1186/1477-7525-11-100

**Published:** 2013-06-20

**Authors:** Norkhafizah Saddki, Hazama Mohamad, Nor Idayu Mohd Yusof, Dasmawati Mohamad, Norehan Mokhtar, Wan Zaripah Wan Bakar

**Affiliations:** 1 School of Dental Sciences, Universiti Sains Malaysia, Kubang Kerian, Kelantan, 16150, Malaysia; 2 Department of Otorhinolaryngology, School of Medical Sciences, Universiti Sains Malaysia, Kubang Kerian, Kelantan, 16150, Malaysia; 3 Advanced Medical & Dental Institute, Universiti Sains Malaysia, Kepala Batas, Pulau Pinang, 13200, Malaysia

## Abstract

**Background:**

The objective of this study was to determine the validity and reliability of the Malay translated Sleep Apnea Quality of Life Index (SAQLI) in patients with obstructive sleep apnea (OSA).

**Methods:**

In this cross sectional study, the Malay version of SAQLI was administered to 82 OSA patients seen at the OSA Clinic, Hospital Universiti Sains Malaysia prior to their treatment. Additionally, the patients were asked to complete the Malay version of Medical Outcomes Study Short Form (SF-36). Twenty-three patients completed the Malay version of SAQLI again after 1–2 weeks to assess its reliability.

**Results:**

Initial factor analysis of the 40-item Malay version of SAQLI resulted in four factors with eigenvalues >1. All items had factor loadings >0.5 but one of the factors was unstable with only two items. However, both items were maintained due to their high communalities and the analysis was repeated with a forced three factor solution. Variance accounted by the three factors was 78.17% with 9–18 items per factor. All items had primary loadings over 0.5 although the loadings were inconsistent with the proposed construct. The Cronbach’s alpha values were very high for all domains, >0.90. The instrument was able to discriminate between patients with mild or moderate and severe OSA. The Malay version of SAQLI correlated positively with the SF-36. The intraclass correlation coefficients for all domains were >0.90.

**Conclusions:**

In light of these preliminary observations, we concluded that the Malay version of SAQLI has a high degree of internal consistency and concurrent validity albeit demonstrating a slightly different construct than the original version. The responsiveness of the questionnaire to changes in health-related quality of life following OSA treatment is yet to be determined.

## Background

Obstructive sleep apnea (OSA) is a sleep disorder that occurs when breathing is interrupted due to repetitive complete or partial upper airway obstruction [1]. The obstruction is most often due to anatomical and functional abnormalities of the muscles that maintain the airway open during sleep [2]. Narrowing of the airway will impede air flow during breathing, resulting in episodes of heavy snoring, shallow breathing or hypoventilation (hypopnea), cessation of breathing (apnea), and frequent arousals. Definitive diagnosis of OSA can be made by nocturnal polysomnography (PSG) that would demonstrate five or more apnoea-hypopnoea episodes per hour of sleep [1]. The total number of apnoea-hypopnoea episodes per hour of sleep, known as the Apnea Hypopnea Index (AHI), is an index used to assess the severity of sleep apnea which can be classified as mild (5 to 15 episodes per hour), moderate (15 to 30 episodes per hour), and severe (greater than 30 episodes per hour) [1].

Often, patients are not aware of their heavy snoring and arousals although the episodes may occur repeatedly up to hundreds of times each night [3]. Multiple nocturnal arousals with sleep fragmentations may lead to excessive daytime sleepiness, fatigue, and impaired concentration in patients with OSA [4]. It was reported that at least 9-15% of middle-aged adults were affected although many cases remain undiagnosed and the prevalence of OSA may have been largely underestimated [5]. The impact of OSA is potentially substantial with extensive evidence on its association with reduced physical, emotional, and intellectual abilities, cardiovascular and cerebrovascular morbidity and mortality, high medical care costs, and road safety risk [6-8]. The quality of life of patients with OSA is often impaired [9,10], in addition to their bed partners whose sleep would also be disrupted due to the patients’ heavy snoring, gasping, and choking, or because of the bed partners’ own concern about the patients’ abnormal breathing [11].

Given the detrimental impact of OSA on individual health and well-being, assessment of health-related quality of life (HRQOL) in patients with OSA is receiving increased attention in clinical practice and research. Generic instruments such as the Medical Outcome Study Short Form (SF-36), Sickness Impact Profile scale, Nottingham Health Profile, Functional Limitations Profile, WHOQOL-BREF and EuroQol have been used to measure HRQOL in patients with sleep disorders [12]. Although generic instruments are applicable to most people irrespective of the type of illnesses by examining a wide range of activities and roles, they may not be sensitive to certain problems that are unique to a particular disease or condition.

Few OSA-specific HRQOL instruments have been developed to measure specific impact of the disease. Such instruments include the Sleep Apnea Quality of Life Index (SAQLI) [13], the Functional Outcomes of Sleep Questionnaire (FOSQ) [14], the Obstructive Sleep Apnea Patient-Oriented Severity Index (OSAPOSI) [15] and the Quebec Sleep Questionnaire (QSC) [16]. Systematic review and content comparison of various OSA-specific quality of life instruments highlighted the usefulness of SAQLI in evaluating patients’ responses to clinical treatments [10,17]. While clinical parameters such as the AHI and subjective symptoms such as daytime fatigue and excessive sleepiness as well as nighttime snoring may improve with treatment, HRQOL may not, and the reverse may also be true. There is a need for an instrument that can detect changes in the quality of life of patients following treatment interventions, and SAQLI has this advantage over other instruments [17]. Therefore, we conducted this study to validate a Malay version of the SAQLI in patients with OSA and this paper reports the preliminary psychometric properties of the Malay version of SAQLI administered to OSA patients prior to their intervention treatments.

## Methods

### Population and sample

This cross sectional study was conducted from March 1, 2010 to March 31, 2011. Source population consisted of patients with sleep-related breathing problems who were referred to the OSA Clinic at the Department of Otorhinolaryngology-Head and Neck Surgery, Hospital Universiti Sains Malaysia. After initial clinical assessments, patients with symptoms suggestive of OSA were subjected to a full-night polysomnography. Patients aged 18 years and above who demonstrated five or more apnoea-hypopnoea episodes per hour of sleep were considered eligible for this study. Systematic random sampling method was applied for selection of study sample. No possible biases regarding the selection of sample were anticipated and the sample was representative of the reference population. Ethical approval was obtained from the Human Research Ethics Committee, Universiti Sains Malaysia.

### Research instrument – the Sleep Apnea Quality of Life Index (SAQLI)

The SAQLI is an OSA-specific questionnaire developed by Flemons and Reimer [13] as a comprehensive HRQOL measure for use in clinical trials. The questionnaire was developed in English and has since been translated into few other languages including Chinese, French, Lithuanian, Spanish and Portuguese. In general, the original English version and the translated versions of SAQLI have been shown to be valid and reliable to measure HRQOL in patients with OSA [18-23]. The SAQLI items were organised into 5 domains to measure the impact of OSA on patients’ daily functioning (domain A), social interactions (domain B), emotional functioning (domain C), symptoms (domain D), and treatment-related symptoms (domain E). There were 11 items in domain A, 13 items in domain B, and 11 items in domain C. Domain D listed more than 20 OSA symptoms and patients may include additional symptoms they may experience. However, patients were required to choose and rate only 5 symptoms most important to them. Domain D was thus considered a 5-item domain although patients may select fewer than 5 symptoms or even none. Similarly, in domain E, patients who have been treated for OSA were asked to identify only the 5 most important symptoms, or less, from the list of symptoms provided, and patients may also add other symptoms to the list. Domain E was considered optional, only to be used in clinical interventions to measure changes in HRQOL following treatment.

The SAQLI was designed to be interviewer-administered and the reference period was 4 weeks prior to the date of interview. Responses for the items were scored on a 7-point Likert scale that ranged from 1 (greatest impairment) to 7 (least impairment). For each domain A, B and C, the mean domain score is derived by dividing the sum of scores for all items within the particular domain by the number of items. For domain D, the sum of scores is divided by 5 regardless of the number of symptoms selected by patients. To obtain the total pre-intervention SAQLI score, the mean scores of 4 domains, domain A, B, C and D, are summed and divided by 4.

If domain E is used following intervention, each score given by patients to all items must first be re-coded before the mean domain score can be obtained in a similar manner used for domain D. Additionally, there is section F that must be completed when domain E is used. This section assesses: 1) the impact of treatment on patients’ overall quality of life as reflected by items in domain A, B, C and D, and 2) the impact of treatment-related symptoms on their quality of life. Two 10 cm visual analogue scales were used to measure the impacts and patients will be asked to mark a vertical line on the individual scales. The lengths of both marks will be determined and recorded as the impact scores. A weighting factor is then obtained by dividing the impact score for treatment-related symptoms by the impact score for quality of life improvements in domain A, B, C and D. This weighting score must be multiplied by the previously obtained mean score for domain E. The product is then subtracted from the total mean scores from the first four domains, and the resulting score is divided by 4 to obtain the total post-intervention SAQLI score. The score for each SAQLI domain and the total SAQLI scores may range from 1 to 7, with higher score indicating better HRQOL.

### Translation and adaption of the Malay version of SAQLI

The translation and adaptation of the SAQLI into Malay language follows the methods adapted by the International Quality of Life Assessment (IQOLA) Project Group [24]. The forward translation of the English version of SAQLI was performed independently by two native Malay speakers who were also fluent in English. One of the translators was a linguist and another was a medical physician with experience in managing OSA. Both forward translations were revised by the translators who then agreed upon one version. The chosen forward-translated version was critically appraised by the researchers before it was passed to two other bilingual translators for quality rating. Feedbacks from the quality raters were reviewed and necessary modifications were made to the items.

Next, another pair of bilingual native speakers translated the forward-translated version back into English. The backward translation processes were also done independently. Finally, both back-translations were reviewed for equivalences with the original English version. Discrepancies between the back-translations, the English version, and the forward-translated version were reconciled to produce a preliminary Malay version of SAQLI. Content validity of the Malay version of SAQLI was also reviewed. The preliminary Malay version of SAQLI was pre-tested on 5 conveniently selected patients with OSA. In general, all respondents could easily understand the preliminary version of the questionnaire. Only minor formatting problems were noted and appropriate modifications were made.

### Data collection

The final Malay version of SAQLI was administered to 82 OSA patients. In addition, the patients also completed the Medical Outcomes Study Short Form (SF-36). This was to test concurrent validity of the Malay-translated SAQLI by comparing its correlation with a more established instrument like the SF-36 which is perhaps the most widely used HRQOL instrument and is considered the gold standard in measure of health status. The SF-36 is also the most frequently used generic HRQOL instrument in OSA [10]. The 36-item instrument was developed to measure 8 health concepts; physical functioning (limitations in physical activities because of health problems), role-physical (limitations in usual role activities because of physical health problems), bodily pain, general health, vitality (energy and fatigue), social functioning (limitations in social activities because of physical or emotional problems), role-emotional (limitations in usual role activities because of emotional problems), mental health (psychological distress and well-being), and general health perceptions [25]. Items in the role-physical and role-emotional use ‘yes/no’ answers while other items are scored on a 3- to 6-point Likert scale. For each item, the raw score were coded, 10 of which were re-coded in the opposite direction, summed, and transformed according to the standard SF-36 scoring algorithm [26]. Each dimension is reported on a scale of 0 (worst possible health state) to 100 (best possible health state). The previously validated Malay version of SF-36 was used in this study [27]. Both questionnaires were interviewer-administered. The Malay version of SAQLI was re-administered to 23 patients after one to two weeks to assess the reliability of scores. Written informed consent was obtained from all patients prior to administration of the questionnaires.

Continuous positive airway pressure (CPAP) is currently the first choice therapy for OSA. It is a medical device that provides continual positive air pressure via a mask to help keep a patient’s airway open during sleep and has been proven to be effective in reducing respiratory disturbances and symptoms of sleepiness [28]. The CPAP machines however are expensive. In our setting, not many patients can afford to buy the machine and most diagnosed OSA patients will opt for other treatment options including lifestyle modifications such as weight loss, regular exercise and sleep repositioning, and a range of upper airway surgical procedures. Owing to this technical constraint in providing the mainstay of OSA treatment to the patients, we thus decided not to use the fifth SAQLI domain in this study. Only domain A, B, C and D were included in this psychometric evaluation of the Malay version of SAQLI.

### Statistical analysis

Data entries and analyses of results were done using the Predictive Analytics Software (PASW) for Windows, version 18.0 (SPSS Inc, Chicago). Descriptive statistics of categorical and numerical data were presented by frequencies and means respectively. Exploratory factor analysis was applied to examine the construct validity of the Malay version of SAQLI. The Kaiser-Meyer-Olkin Measure of Sampling and the Bartlett's Test of Sphericity were first performed to determine the suitability of data for factor analysis. The factor analysis was then conducted using principle component analysis at an eigenvalue of 1.0 with an orthogonal (varimax) rotation solution. Independent *t*- test was used to compare the HRQOL constructs of the Malay version of SAQLI between participants with different category of OSA severity. The internal consistency reliability of the factors was evaluated using Cronbach’s alpha. Test-retest reliability was evaluated using intraclass correlation coefficient (ICC). The concurrent validity of the Malay version of SAQLI was examined by determining the Pearson’s correlation coefficients between the SAQLI domains and the Malay-translated SF-36 scales. The results were interpreted following the recommendations provided by Colton et al. [29], correlation coefficients between 0 and 0.25 (or −0.25) indicate little or no relationship; from 0.25 to 0.50 (or −0.25 to −0.50) a fair degree of relationship; from 0.50 to 0.75 (or −0.50 to −0.75) a moderate to good relationship; and between 0.75 and 1 (or −0.75 and −1) a very good to perfect relationship.

## Results

### Descriptive statistics

Characteristics of the participants are shown in Table 1. The participants were 69.5% male and the mean age was 39.6 years (SD 12.82). Their Body Mass Index (BMI) was classified into normal (18.5-24.99), pre-obese (25.00-29.99), obese class I (30.00-34.99), obese class II (35.00-39.99) and obese class III (≥40.00) [30]. Most of the participants (46.3%) were considered obese with BMI of 30.00 or greater. There was a wide range of sleep apnea severity with AHI ranging from 5.5 to 97.0. Most participants had AHI greater than 30, indicating severe OSA (51.2%) with a mean AHI of 33.9 (SD 21.69).

**Table 1:** **Characteristics of patients** (**n** = **82**)

**Characteristic**	**Frequency (%)**
Sex	
Male	57 (69.5)
Female	25 (30.5)
Age group (years)	
≤ 24	13 (15.9)
25 – 34	20 (24.4)
35 – 44	19 (23.2)
≥ 45	30 (36.6)
Body Mass Index	
18.50-24.99	12 (14.6)
25.00-29.99	32 (39.0)
30.00-34.99	23 (28.0)
35.00-39.99	9 (11.0)
≥40.00	6 (7.3)
OSA severity	
Mild	15 (18.3)
Moderate	25 (30.5)
Severe	42 (51.2)

### Construct validity of the Malay version of SAQLI

The Kaiser-Meyer-Olkin value was high at 0.929 and the Bartlett’s Test of Sphericity was significant (*p* < 0.001), confirming the appropriateness of using factor analysis on the data. The communality values of all items were above 0.5 which indicate that each item shared some common variance with other items. Forty items were analysed and four factors with eigenvalues above 1 were extracted, which accounted for 81.14% of the variance. With a 0.50 cut point for inclusion of an item in interpretation of factors, all 40 items loaded higher than the set threshold. However, the factor loadings of the items were not entirely consistent with the proposed construct. Although the majority of items in domain A (daily functioning), domain B (social interactions), and domain C (emotional functioning) loaded together appropriately in accordance with the proposed subscale, items in domain D (symptoms) behaved somewhat different. Instead of loading into one factor, all items in domain D were combined with items in domain A to load on the first factor. This first factor, which also covered one item in domain B (B7: looked for excuses for being tired), has a total of 17 items and accounted for 66.21% of the total variance. The second factor covered 12 of 13 items in domain B and explained 6.96% of the total variance. Items in domain C were fragmented, nine loaded on the third factor (C1-C9) and two other items (C10-C11) loaded on the fourth factor. The third factor accounted for 5.00% of the total variance and the fourth factor which was the smallest factor accounted for 2.96% of the total variance.

According to Costello and Osborne [31], a solid factor is a factor with 5 or more items with strong loadings of at least 0.5. On the other hand, a factor with less than three items is considered weak and unstable. While Factor 1, 2 and 3 each has more than the recommended number of items, Factor 4 has only two items loading. However, both items have high loadings above 0.63 which can be classified as very good loadings [32]. The communalities of both items were also high at 0.872 and 0.830 for item C10 and item C11 respectively. Thus, we decided to keep both items as they were important items that relate patients concern about their weight (C10) and heart problems and/or premature death (C11). The factor analysis was repeated on all 40 items with a forced three factor solution. This resulted in Factor 1 containing 13 items (explaining 66.21% of the variance), Factor 2 containing 18 items (explaining 6.96% of the variance) and Factor 3 containing the remaining 9 items (explaining 5.00% of the variance). Variance accounted by the three factors was 78.17%. All items had primary loadings over 0.55. The factor loading matrix for this final solution is presented in Table 2. Similar with previous rotation, the majority of items in domain A (daily functioning), domain B (social interactions), and domain C (emotional functioning) loaded together appropriately while items in domain D (symptoms) were combined with items in domain A. Accordingly, the original factor labels, daily functioning, social interactions, and emotional functioning, were retained to describe the extracted factors in the new construct. Table 3 shows the descriptive statistics of the Malay version of SAQLI. All SAQLI domains had no floor or ceiling effects.

**Table 2:** **Communalities** (***h***^***2***^) **and factor loadings by Varimax rotation with Kaiser normalization**

**Domain**/**item**	**Factor 1**	**Factor 2**	**Factor 3**	***h***^***2***^
	**Social interactions**	**Daily functioning**	**Emotional functioning**	
A: daily functioning				
A1		**0**.**751**		0.826
A2		**0**.**799**		0.705
A3		**0**.**769**		0.789
A4		**0**.**688**		0.754
A5		**0**.**823**		0.805
A6		**0**.**719**		0.774
A7		**0**.**822**		0.795
A8		**0**.**757**		0.711
A9		**0**.**621**		0.621
A10		**0**.**637**		0.666
A11		**0**.**780**		0.859
B: social interactions				
B1	**0**.**651**			0.687
B2	**0**.**661**			0.736
B3	**0**.**793**			0.858
B4	**0**.**842**			0.872
B5	0.591	**0**.**623**		0.739
B6	**0**.**870**			0.898
B7		**0**.**677**		0.819
B8	**0**.**768**			0.865
B9	**0**.**783**			0.858
B10	**0**.**830**			0.887
B11	**0**.**792**			0.861
B12	**0**.**731**			0.826
B13	**0**.**804**			0.860
C: emotional functioning				
C1	**0**.**625**		0.599	0.825
C2			**0**.**602**	0.718
C3	0.603		**0**.**638**	0.840
C4	0.537		**0**.**694**	0.868
C5	0.579		**0**.**665**	0.853
C6	**0**.**674**			0.776
C7	0.617		**0**.**619**	0.794
C8	0.592		**0**.**626**	0.841
C9			**0**.**609**	0.680
C10			**0**.**690**	0.571
C11			**0**.**635**	0.608
D: symptoms				
D1		**0**.**650**	0.554	0.838
D2		**0**.**628**		0.803
D3		**0**.**684**		0.749
D4		**0**.**692**		0.762
D5		**0**.**607**		0.672

Note: Values 0.55 and lower were suppressed.

**Table 3:** Descriptive statistics of the Malay version of SAQLI

**Instrument/domain**	**Mean (SD)**	**Range**	**% Floor**	**% Ceiling**
Malay version of SAQLI				
Daily functioning	3.9 (0.87)	2.1-5.7	0.0	0.0
Social interactions	4.9 (0.95)	2.7-6.1	0.0	0.0
Emotional functioning	4.4 (0.84)	2.2-5.6	0.0	0.0
Total scale	4.4 (0.83)	2.4-5.7	0.0	0.0

Note: SAQLI domain score ranges from 1 to 7.

In addition to factor analysis, extreme group comparisons were also performed in determining the construct validity of the Malay version of SAQLI. Table 4 shows the mean SAQLI scores of participants grouped by OSA severity assessed by the AHI. As the number of participants with mild OSA was quite small (n = 15), regrouping was done to combine the mild and moderate OSA into one category. Significantly lower mean SAQLI scores were observed in participants with mild or moderate OSA compared to those with severe OSA in all subscales of the Malay version of SAQLI.

**Table 4:** Mean scores of the Malay version of SAQLI by OSA severity

**Instrument/domain**	**OSA**		**t statistic (d.f.)**	***p *value**
	**Mild/moderate**	**Severe**		
	**(n = 40)**	**(n = 42)**		
	**Mean (SD)**	**Mean (SD)**		
Malay version of SAQLI				
Daily functioning	4.3 (0.73)	3.4 (0.79)	5.12 (80)	<0.001
Social interactions	5.3 (0.75)	4.6 (0.98)	3.89 (80)	<0.001
Emotional functioning	4.7 (0.72)	4.1 (0.84)	3.49 (80)	0.001
Total scale	4.8 (0.69)	4.1 (0.79)	4.49 (80)	<0.001

### Concurrent validity

Table 5 shows the correlation between the Malay version of SAQLI and SF-36 domains. Significant correlations observed between all SAQLI and SF-36 domains (p < 0.001) showed evidence of concurrent validity. The correlation coefficients ranged from 0.713 to 0.916, suggesting positive and good to excellent correlations. The daily functioning domain of the Malay version of SAQLI showed the highest correlation with the vitality scale of SF-36 with correlation coefficient of 0.916. Meanwhile, the social interactions domain and the emotional functioning domain of SAQLI had the highest correlation with the mental health scale of SF-36 with correlation coefficients of 0.850 and 0.845 respectively. The total SAQLI score also showed the highest correlation with the mental health scale (r = 0.907).

**Table 5:** **Correlation between the Malay version of SAQLI and SF**-**36 domains using Pearson**’**s correlation coefficient**

**Instrument/domain**	**Malay version of SAQLI**			
	**Daily functioning**	**Social interactions**	**Emotional functioning**	**Total scale**
SF-36				
Physical functioning	0.866	0.759	0.737	0.841
Role-physical	0.858	0.713	0.771	0.832
Bodily pain	0.875	0.778	0.755	0.858
General health	0.885	0.763	0.747	0.852
Vitality	**0**.**916**	0.825	0.778	0.898
Social functioning	0.790	0.754	0.783	0.828
Role-emotional	0.844	0.715	0.766	0.827
Mental health	0.850	**0**.**850**	**0**.**845**	**0**.**907**

Note: All correlations are significant (*P* < 0.001).

### Internal consistency and test-retest reliability

Table 6 shows results of internal consistency reliability analysis of the Malay version of SAQLI. The Cronbach’s alpha values were very high for all domains; daily functioning 0.978, social interactions 0.980, and emotional functioning 0.947. The Cronbach’s alpha value for the whole scale was 0.987. The corrected item-total scale correlations ranged from 0.752 to 0.916 for the daily functioning domain, 0.778 to 0.938 for the social interactions domain, and 0.551 to 0.913 for the emotional functioning domain.

**Table 6:** Internal consistency reliability of the Malay version of SAQLI

**Domain/item**	**Corrected item-total correlation**	**Cronbach’s alpha if item deleted**	**Cronbach’s alpha for domain**
Daily functioning (18 items)			0.978
A1	0.885	0.977	
A2	0.782	0.978	
A3	0.869	0.977	
A4	0.854	0.977	
A5	0.859	0.977	
A6	0.856	0.977	
A7	0.852	0.977	
A8	0.794	0.978	
A9	0.760	0.978	
A10	0.795	0.978	
A11	0.916	0.976	
B5	0.752	0.978	
B7	0.882	0.977	
D1	0.869	0.977	
D2	0.861	0.977	
D3	0.838	0.977	
D4	0.852	0.977	
D5	0.793	0.978	
Social interactions (13 items)			0.980
B1	0.802	0.979	
B2	0.837	0.979	
B3	0.917	0.977	
B4	0.917	0.977	
B6	0.919	0.977	
B8	0.914	0.977	
B9	0.914	0.977	
B10	0.938	0.977	
B11	0.916	0.977	
B12	0.892	0.978	
B13	0.913	0.977	
C1	0.797	0.980	
C6	0.778	0.980	
Emotional functioning (9 items)			0.947
C2	0.794	0.940	
C3	0.886	0.935	
C4	0.913	0.933	
C5	0.887	0.935	
C7	0.851	0.937	
C8	0.890	0.935	
C9	0.776	0.941	
C10	0.551	0.951	
C11	0.538	0.952	
Total scale			0.987

We noted that the Cronbach’s alpha would increase if item C10 (concern about weight) and C11 (concern about heart problems and/or premature death) were deleted from the emotional functioning domain. However, deletion of any of these items will not result in enhanced reliability of the instrument as the increase in Cronbach's alpha would only be very minimal, from 0.947 to 0.951 and 0.952 for C10 and C11 respectively. Besides, both items correlate fairly well with the composite score, the corrected item-total correlations for item C10 and C11 were 0.551 and 0.538 respectively. Thus there is no valid statistical reason to drop the items and we maintained both items in the scale. The loadings of C10 and C11 in the final 3-factor solution were also high at 0.690 and 0.635 respectively (Table 2).

Test-retest reliability analysis was done on 23 patients. All ICC were significant (*P* < 0.001). The ICC were 0.982 (95% CI 0.958, 0.992) for daily functioning domain, 0.985 (95% CI 0.966, 0.994) for social interactions domain, and 0.975 (95% CI 0.943, 0.989) for emotional functioning domain.

## Discussion

The SAQLI questionnaire was developed specifically to determine HRQOL in patients with OSA [13]. The present study is among the few studies that explored the factor structure of SAQLI. To our knowledge, only three previous studies had performed factor analysis of SAQLI. Those were studies that validated the Chinese version [18], the Portuguese version [22], and the Spanish version of SAQLI [21]. In other versions of SAQLI, namely the French version [19], the Lithuanian version [20], and the original English version by Flemons and Raimer [13], the construct validity of the respective questionnaires was determined based on their positive correlations with other comparable instruments like the SF-36 and/or the ability to successfully discriminate HRQOL of OSA patients before and after treatment.

Results of our factor analysis showed that some items of the Malay version of SAQLI did not load on the theorised factor structure. Similarly, the Chinese version of SAQLI showed pattern of item loadings that were different from the original version [18]. The constructs of the Portuguese and the Spanish versions of SAQLI however were considered comparable with the hypothesised scales by their respective authors although the loading of items on the factors were not entirely similar with original domains [21,22].

While previous studies had maintained four factors from the items of their respective versions [18,21,22], we concluded only three domains from the Malay version of SAQLI. As highlighted in the results, all items in the symptoms domain were combined with items in the daily functioning domain instead of loading on their individual factor. This has not been reported before but it is a plausible finding because the listed symptoms of OSA were closely associated with impacts on daily activities, for example items like “Feeling that ordinary activities require an extra effort to perform or complete”, “Difficulty staying awake while reading”, and “Fighting the urge to fall asleep while driving”. In addition, item B5 (need to make special sleeping arrangements if you were travelling and/or staying with someone) and B7 (looked for excuses for being tired) in the social interactions were also collected with items of the daily functioning and the symptoms domain. The loading of B5 on daily functioning domain was in agreement with Mok et al. [18] while the loading of B7 was in agreement with Sampaio, et al. [22]. All items in the daily functioning domain were collected together with loadings that were considered good (0.55 or higher), very good (0.63 or higher) or excellent (0.71 or higher) based on the classification by Comrey and Lee [32]. These include item A6 (finding the time for activities that you find relaxing) and A9 (trying to remember things) which were considered problematic in other versions. Item A6 was poorly loaded in both the Chinese and Spanish versions of SAQLI while item A9 was poorly loaded in the Portuguese version. Another noteworthy finding on the construct of the Malay version of SAQLI was the combination of emotional functioning items (C1: depressed, down and/or hopeless and C6: being unreasonable) with items in the social interactions domain. This was in agreement with the results reported in the Chinese, Portuguese and Spanish versions [18,21,22].

During development of the original SAQLI, item reduction was performed by means of clinical impact analysis which ranked the most frequent and the most important items rated by patients [13]. The selected items were then categorised into domains based on clinical sensibility. In this study, as well as in studies by Catalán et al. [21], Mok et al. [18], and Sampaio et al. [22], the SAQLI items were grouped into different domains using factor analysis approach that examines the structure of correlations between items. Owing to the different item selection approaches used, dissimilarities found between the construct of the Malay translated and the original version of SAQLI thus did not come as a surprise. It has been demonstrated in a study by Juniper et al. [33] that the use of different item reduction methods resulted in selection of different items into different domains even to an extent that appreciably different instruments might be produced. In addition, these deviations from the hypothesised loadings may also be a reflection of how health and aspects of well-being were understood and expressed by patients in Malaysia.

Culture-related factors have been found to influence HRQOL dimensions, and what is perceived as an important HRQOL aspect by people in one culture may not be as important to people in other cultures [34]. The different ways that the concept of HRQOL was perceived by people in Malaysia compared to the Westerners have been demonstrated in previous studies [27,35,36]. Nevertheless, researchers have been strongly cautioned against modifying a validated questionnaire since even small changes can destroy its validity [37]. Hence, cross-cultural adaptation of a validated questionnaire for use in other cultures and/or languages requires a methodical process in order to achieve equivalences between the original version and the translated version, and to maintain the content validity of the instrument across different cultures [24]. The Malay version of SAQLI used in this study has not been modified in any way. All original items, responses, and formatting were maintained, and the cultural adaptation process was done by experts following guidelines recommended by the IQOLA Project Group [24]. Although the Malay version of SAQLI demonstrated a different construct than the original English version, there were more similarities than divergence between the two, which added credence to the construct validity of the Malay version of SAQLI. Nevertheless, future studies are recommended to explore the consequences of rearranging items of the Malay version of SAQLI into the new construct.

The Malay version of SAQLI demonstrated excellent internal consistency. The Cronbach’s alpha for all domains were well over 0.70, the minimum threshold acceptable in most social science research situations [38]. In addition, each item had a strong, positive corrected item-total correlation which indicates each item correlates well with other items in its respective domain. It is also evident that each item was useful and contributed to the overall reliability of the domain. Similar findings were obtained in all other versions of SAQLI [18-23]. Nevertheless, these very high Cronbach’s alpha reliability coefficients should be interpreted with caution. Although the higher Cronbach’s alpha is generally the better because it indicates a more reliable scale, a too high value may not always be desirable. According to Streiner [39], the Cronbach’s alpha values that are higher than 0.90 may reflect unnecessary duplication of items which will result in a needlessly long questionnaire. Hence, we are in total agreement with Lacasse et al. [19] Mok et al. [18] and Sampaio et al. [22] that there could be a shorter version of SAQLI, not only with fewer items but also perhaps with fewer domains. Further, there is a practical need to have a shorter version of SAQLI because a long questionnaire that takes too much time to complete may not be readily accepted by both patients and health care providers in busy clinical settings.

The Malay version of SAQLI was able to differentiate between patients with mild/moderate OSA and patients with severe OSA. The mean scores of patients with mild/moderate OSA were significantly higher compared to patients with severe OSA in all domains. These findings support the usefulness of SAQLI in detecting differences in HRQOL across different levels of OSA severity. In previous studies, the instrument has been shown to be responsive to changes in HRQOL of OSA patients following therapeutic interventions [18-23]. Discriminative capability is an important well-known feature of SAQLI [17].

Positive significant interdomain correlations were observed between the Malay version of SAQLI and the Malay version of SF-36, and the highest correlations were found between comparable domains. The daily functioning domain of the Malay version of SAQLI showed the highest correlation with the vitality scale of SF-36 while maintaining very good to perfect correlation with other SF-36 domains. In addition, the social interactions domain and the emotional functioning domain of the Malay version of SAQLI had the highest correlation with the mental health scale of SF-36 while maintaining moderate to good correlations with other SF-36 domains. Similarly, our total SAQLI score also showed the highest correlation with the mental health scale while keeping very good to perfect correlation with other SF-36 domains. Collectively, these results provide strong evidence for concurrent validity of the Malay version of SAQLI. Significant interdomain correlations were also obtained by other authors who used the SF-36 for testing the concurrent validity of their respective versions of SAQLI [18,19,21,23]. Sampaio et al. [22] used the Hospital Anxiety and Depression Scale for testing the concurrent validity of the Portuguese version of SAQLI and negative interdomain correlations were obtained between the SAQLI domain and the anxiety and depression scales.

In more recent times, HRQOL assessment has become an important outcome indicator in evaluation of clinical treatments of OSA alongside the conventional clinical, physiological and laboratory parameters [40]. Valid, reliable, and responsive instruments should thus be available. In this paper, the psychometric properties of the Malay version of SAQLI administered to OSA patients prior to their intervention treatments were reported. Our sample size was adequate for factor analysis based on the recommendations by MacCallum et al. [41]. Earlier, several guidelines on sample size in factor analysis have been proposed. These include recommmendations based on minimum necessary sample size such as 100 [42], 300 [43], and 500 [32] or based on minimum subject-to-variable ratio which may range from as low as 2:1 [44] to as high as 20:1 [45]. However, MacCallum et al. [41] had defied these general rules of thumb on minimum sample size for factor analysis. Instead, according to MacCallum et al. [41], judgement of sample size adequacy can be made after the analysis as it depends more on data characteristics such as communalities and factor-to-variable ratio. In the present study, we achieved 3 factors with 40 items, representing a high variable-to-factor ratio. The communalities were consistently high and the mean level of communality was 0.78 (SD 0.08), higher than the recommended threshold of 0.70 [41]. Thus, we are confident that the resulting factors correspond closely to the population factors, even with moderate sample size.

## Conclusions

Our preliminary results showed that the Malay version of SAQLI is a valid and reliable instrument for measuring HRQOL in OSA patient although there may be some redundancy in items. Thus, we recommend for a shorter version of SAQLI which undoubtedly will be more practical for use in busy clinical settings. In addition, more studies are required to explore the factor structure of SAQLI in different population settings.

## Competing interests

The authors declare that they have no competing interests.

## Authors’ contributions

All authors contributed to the conception and design of the study. NIMY and NKS contributed to data acquisition, management, and analyses. All authors contributed to data interpretation. NKS contributed to preparation of the manuscript. All authors revised and approved the final manuscript.
